# Associations between PBMC whole genome transcriptome, muscle strength, muscle mass, and physical performance in healthy home-dwelling older women

**DOI:** 10.1007/s11357-023-00819-0

**Published:** 2023-05-19

**Authors:** Ana R. S. de Sousa, Inger Ottestad, Gyrd O. Gjevestad, Kirsten B. Holven, Stine M. Ulven, Jacob J. Christensen

**Affiliations:** 1 Department of Nutrition, Institute of Basic Medical Sciences, University of Oslo, Sognsvannsveien 9, 0372 Oslo, Norway; 2 The Clinical Nutrition Outpatient Clinic, Section of Clinical Nutrition, Department of Clinical Service, Division of Cancer Medicine, Oslo University Hospital, Sognsvannsveien 20, 0372 Oslo, Norway; 3 TINE SA, Innovation and Marketing, Postboks 113 Kalbakken, 0902 Oslo, Norway; 4 Norwegian National Advisory Unit On Familial Hypercholesterolemia, Department of Endocrinology, Morbid Obesity and Preventive Medicine, Oslo University Hospital, Forskningsveien 2B, 0373 Oslo, Norway

**Keywords:** CIBERSORT, Gene expression, Muscle, Older adults, PBMCs, WGCNA

## Abstract

Increasing age is accompanied by many changes, including declining functional skeletal muscle health and immune dysfunction. Peripheral blood mononuclear cells (PBMCs) are circulating cells that assemble an immune response, but their whole genome transcriptome has not been studied in the context of age-related muscle health. Consequently, this article explored associations between three muscle variables indicative of functional muscle health — maximum handgrip strength (muscle strength), appendicular skeletal muscle mass index (ASMI, muscle mass), and gait speed (physical performance) — and two groups of bioinformatics-generated PBMC gene expression features (gene expression–estimated leukocyte subset proportions and gene clusters). We analyzed cross-sectional data from 95 home-dwelling healthy women ≥ 70 years, using “cell-type identification by estimating relative subsets of RNA transcripts” (CIBERSORT) to estimate leukocyte subset proportions and “weighted correlation network analysis” (WGCNA) to generate gene clusters. Associations were studied using linear regression models and relevant gene clusters were subjected to gene set enrichment analysis using gene ontology. Gait speed and ASMI associated with CIBERSORT-estimated monocyte proportions (*β* =  − 0.090, 95% CI = (− 0.146, − 0.034), *p*-value = 0.002 for gait speed, and *β* =  − 0.206, 95% CI = (− 0.385, − 0.028), *p*-value = 0.024 for ASMI), and gait speed associated with CIBERSORT-estimated M2 macrophage proportions (*β* =  − 0.026, 95% CI = (− 0.043, − 0.008), *p*-value = 0.004). Furthermore, maximum handgrip strength associated with nine WGCNA gene clusters, enriched in processes related to immune function and skeletal muscle cells (*β* in the range − 0.007 to 0.008, *p*-values < 0.05). These results illustrate interactions between skeletal muscle and the immune system, supporting the notion that age-related functional muscle health and the immune system are closely linked.

**Supplementary Information:**

The online version contains supplementary material available at 10.1007/s11357-023-00819-0.

## Introduction

Increasing age is accompanied by changes in skeletal muscle tissue. These changes are experienced by all individuals, even healthy, physically active, and well-nourished older adults [1]. Aging skeletal muscle exhibits altered parameters indicative of functional skeletal muscle health, such as reduced skeletal muscle mass (as reviewed in [2]), strength [3], and physical performance [3, 4]. Reduced functional muscle health is associated with a series of negative outcomes in the older population, such as increased risk of falls, inability to perform daily activities, cognitive impairment, and lower quality of life (as reviewed in [5]). Functional muscle health metrics have been shown to be of importance for both physical and mental quality of life, in older individuals [6].

Increasing age also encompasses changes in the immune system. The aging innate immune system is chronically activated, leading to the development of chronic low-grade inflammation, for which monocytes are essential (reviewed in [7]). More specifically, in older adults, monocytes seem to spontaneously secrete altered amounts of inflammatory cytokines [8] and fail to efficiently resolve inflammatory insults [9]. Age also affects the adaptive immune system, resulting in lower proportions of naïve cells, an accumulation of effector and memory cells, and impaired function of lymphocytes, among others (reviewed in [10]). Overall, these alterations lead to a dysregulation of the immune system in older adults, exemplified by poor response to vaccines [11], high incidence of chronic conditions (including diabetes, heart disease, and cancer) [12], and serious infections [13].

Muscle health and the immune system are linked, which is evident in healthy muscle tissue, but also in various conditions characterized by reduced muscle health. For example, in healthy skeletal muscle, inflammatory mediators modulate muscle protein metabolism [14], and the action of resident and infiltrating immune cells from the circulation is central for muscle healing [15]. In older individuals with frailty syndrome, the frailest have the highest levels of circulating inflammation markers [16] and increased numbers of circulating monocytes [17].

Easily accessible peripheral blood mononuclear cells (PBMCs) can be used to assess the immune system [18]. Via blood, circulating immune cells are transported between organs, migrate from site to site, and are exposed to systemic factors, being able to provide a snapshot of the immune networks at work in the body. This snapshot can be acquired through genomic approaches [19]. Gene expression patterns in PBMCs and in skeletal muscle have been reported to be correlated [20], although studies associating age-related functional muscle health and whole genome PBMC gene expression are lacking. Nonetheless, expression of some genes from peripheral blood T-cells and muscle strength have been shown to be associated [21].

Therefore, the main objective of our study was to explore whether variables indicative of functional muscle health (specifically, muscle strength, muscle mass, and physical performance) were associated with whole genome PBMC gene expression features, in previously collected cross-sectional data from Norwegian home-dwelling older adults. Namely, we explored associations between muscle strength (maximum handgrip strength), muscle mass (appendicular skeletal muscle mass index (ASMI)), and physical performance (gait speed) and two groups of bioinformatics-generated PBMC gene expression features (gene expression–estimated circulating leukocyte subset proportions and gene clusters). We hypothesized that functional muscle health was associated with specific circulating leukocyte subset proportions and to immune function, possibly inflammation.

## Methods

### Study design

Between August 2014 and July 2015, home-dwelling older adults (≥ 70 years) residing in Skedsmo (Norway) were recruited to participate in a cross-sectional study, as described in [22]. This study was performed according to the Helsinki Declaration and approved by the Regional Committee for Medical and Health Research Ethics, Health Region South East, Norway (2014/150/REK). Participants were informed about the study and provided their written consent.

### Clinical and biochemical variables

Weight, height, BMI, body composition, information on comorbidities (history of cancer, cardiovascular disease, hypertension, respiratory disease, severe inflammatory disease, and/or type 2 diabetes) and prescribed drugs, Mini Nutritional Assessment (MNA) scores, and Mini-Mental State Examination (MMSE) scores were acquired as described in [21]. The MNA (MNA®-SF) and MMSE (MMSE-2) forms both have a maximum score of 30 points (higher scores are indicative of better nutritional status and cognitive function, respectively). Non-fasting blood samples were taken and analyzed by an accredited laboratory for biochemical parameters [22].

### Muscle strength, muscle mass, and physical performance

Maximum handgrip strength (a measure of muscle strength) was measured in each hand in triplicate, using a digital handheld dynamometer (KE-MAP80K1; Kern Map), as described in [23]. Maximum handgrip strength was considered to be the maximum registered measurement of either hand. Maximum handgrip strength was classified as reduced when < 16 kg [24, 25]. Appendicular skeletal muscle mass was computed summing the muscle mass of both arms and legs measured with bioelectrical impedance analysis. For each participant, appendicular skeletal muscle mass index (ASMI, a measure of muscle mass) was computed by dividing the appendicular skeletal muscle mass by body mass index (BMI). ASMI < 0.512 kg/kg/m^2^ was classified as low [26]. Gait speed (a measure of physical performance) was quantified as part of the Short Physical Performance Battery test, and it was classified as slow if ≤ 0.8 m/s [24].

### Statistical and bioinformatics analyses of PBMC gene expression

From 437 participants, 95 samples were selected for assessment of PBMC gene expression. These samples originated from a selection of age-matched non-smoking older women with and without low muscle mass and/or muscle strength [27]. The gene expression experiments were performed as described in [28], using the Illumina HumanHT-12 v4 Expression BeadChip. After data acquisition, R (http://www.r-project.org) was used to assess the data and to filter out probes, log_2_-transform, and normalize the gene expression data across batches before further analyses [28].

**Fig. 1:**
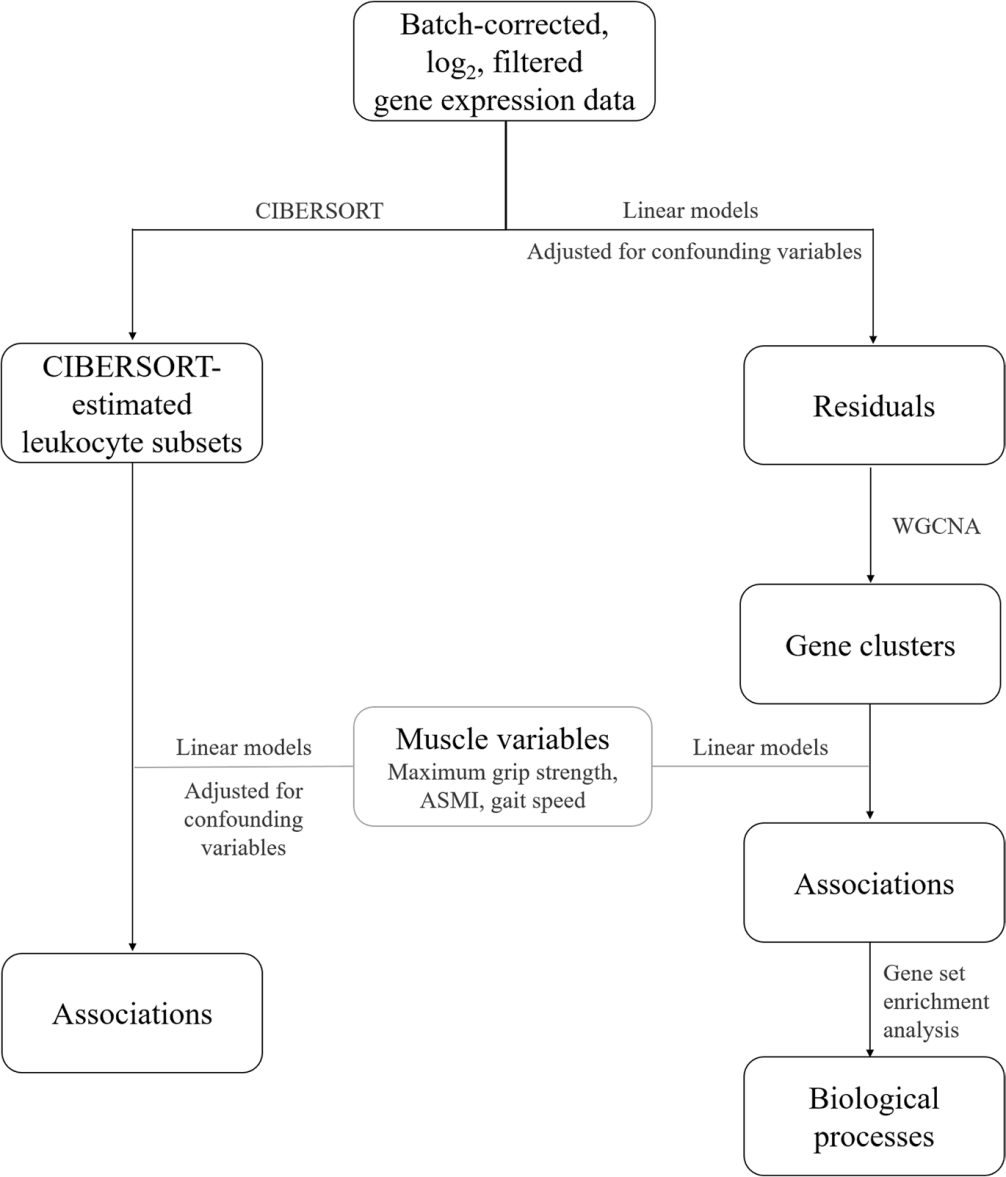
Analysis workflow. The gene expression data were analyzed by two different algorithms: CIBERSORT and WGCNA. The batch-corrected, log_2_-transformed, filtered gene expression data were used directly by the CIBERSORT algorithm, producing an estimate of the proportions of the different leukocyte subsets present in the samples [29]; these results were associated with the muscle variables (ASMI, gait speed, maximum grip strength) using linear regression models adjusted for age and BMI. Prior to WGCNA analysis, the gene expression data were adjusted for age, BMI, and percentage of monocytes and lymphocytes using the residual method. The WGCNA algorithm produced clusters of highly interconnected genes [30], which were associated with the muscle variables with linear regression models. The gene clusters that significantly associated with the muscle variables were subjected to gene set enrichment analysis for gene ontology biological processes

For the present work, we analyzed the data using two different bioinformatics tools, as seen in Fig. 1: “cell-type identification by estimating relative subsets of RNA transcripts” (CIBERSORT) and “weighted correlation network analysis” (WGCNA). R (version 4.1.0) and RStudio IDE (version 1.3.1093) were used to perform the analyses. We computed correlations with Spearman’s rank correlation coefficients (and corresponding *p*-values) using Hmisc::rcorr (read as the “rcorr” function in the “Hmisc” package in R), and sample size for the general linear model (80% power) using pwr::pwr.f2. We considered *p*-values < 0.05 to be statistically significant. Linear regression models were informed by directed acyclic graphs made with PowerPoint® 2016 (Microsoft Office®). Directed acyclic graphs represent causal relationships where an exposure points towards an outcome. Therefore, these graphs help characterize causality and infer logical statistical relations [29]. In the present work, we chose to test the hypothesis of whether aging skeletal muscle is causally associated with PBMC gene expression features, as depicted in Online Supplement 1. It should be noted that, although we tested causal associations, we could conclude on causality, but we could not exclude that associations with the opposing direction are possible.

- CIBERSORT: linear models

CIBERSORT performs *in silico* flow cytometry, using whole genome gene expression data to estimate proportions of cell subsets in a mixed sample, based on reference gene expression signatures for each cell subset [30]. We formatted the gene expression data according to the CIBERSORT requirements and uploaded them onto the CIBERSORT webpage (https://cibersort.standford.edu), where they were analyzed using the reference gene signature LM22 and 100 permutations. CIBERSORT predicts the proportions of 22 leukocyte subsets for each participant sample; for a number of leukocyte subsets, the algorithm estimated proportions of zero for many samples. Therefore, we selected the CIBERSORT-estimated leukocyte subsets with ≥ 65% non-null samples for further analyses: monocytes, T cells CD8, NK cells resting, T cells CD4 naïve, T cells CD4 memory activated, T cells CD4 memory resting, mast cells resting, macrophages M2 (Online Supplement 2).

We computed linear regression models with each of the muscle variables (maximum handgrip strength, ASMI, and gait speed) as exposures and each of the CIBERSORT-estimated leukocyte subset proportions as outcomes, using stats::lm. The linear regression models were adjusted for age and BMI.

b. WGCNA: linear models and gene set enrichment analysis

WGCNA is a systems biology tool that uses weighted correlations between pairs of transcripts to suggest networks and identify clusters of highly interconnected genes [31]. Input of confounded gene expression data into the WGCNA algorithm may result in confounded gene clusters. Therefore, we adjusted the batch-corrected, log_2_-transformed, filtered gene expression data for age, BMI, percentage of monocytes, and percentage of lymphocytes, using a linear regression model. We extracted the residuals and subsequently used them as input for the WGCNA algorithm (Cran, Bioconductor).

We used a soft thresholding power of three, based on plots for scale-free topology model fit and mean connectivity against soft thresholding power, plotted using WGCNA::pickSoftThreshold. WGCNA::blockwiseModules (randomSeed = 1388) produced gene expression clusters in blocks of 5000 gene transcripts using unsigned networks. Clusters with < 20 genes were combined into the gray cluster (minModuleSize = 20). Each cluster was summarized with a cluster eigengene (equivalent to the first principal component) and given a random color name. Cluster eigengenes with correlation coefficients > 0.85 were combined (mergeCutHeight = 0.15). The WGCNA algorithm identified a total of 35 different gene clusters.

We computed linear regression models with each of the muscle variables (maximum handgrip strength, ASMI, and gait speed) as exposures and each of the WGCNA clusters as outcomes, using stats::lm. For each significant linear regression model, we used Gene Ontology (GO) and the biomaRt package to perform gene set enrichment analysis. Ensemble annotations (including GO IDs, gene names, and definitions) were retrieved with biomaRt::useMart (host = “https://nov2020.archive.ensembl.org,” dataset = “hsapiens_gene_ensembl”) and biomaRt::getBM. Using the topGO package, the genes in each cluster were linked to the retrieved annotations by biological process (BP). Enrichment analysis was performed on these objects and summarized with topGO::runTest (algorithm = “classic,” statistic = “fisher”) and topGO::genTable.

## Results

### Study population

Descriptive characteristics from the 95 female participants are summarized in Table 1 and additional information can be found in Online Supplement 3. The participants had a median (p25, p75) age of 77 years (74, 82), BMI of 25.0 kg/m^2^ (22.6, 27.9), HbA1c level of 40 mmol/mol (38, 43), total cholesterol level of 5.6 mmol/L (5.0, 6.3), and none had type 1 diabetes. They had few comorbidities (23% of the participants had ≥ 2 comorbidities) and took few prescription drugs (only 38% of the participants took ≥ 3 drugs/day). The MNA and MMSE scores indicated the participants had good nutritional status (none of the participants had MNA score < 17 points) and good cognitive status (only 6.3% of the participants had an MMSE score < 24 points). Eight participants (8.4%) had reduced maximum handgrip strength (< 16 kg), two participants (2.1%) had reduced ASMI (< 0.502 kg/kg/m^2^), and six participants (6.3%) had reduced gait speed (≤ 0.8 m/s). None of the participants had simultaneous low values for maximum handgrip strength, ASMI, and gait speed, and only two participants had reduced values for two muscle variables simultaneously (maximum handgrip strength and gait speed). The characteristics of the study population by quartiles of maximum handgrip strength, ASMI, and gait speed are found on Online Supplement 4, 5, and 6, respectively.

**Table 1:** Characteristics of the study population. For continuous variables, the values are medians (p25, p75); for categorical variables, the values are n/N. ‡ four missing values; ‡‡ five missing values. Abbreviations: *ASMI*, appendicular skeletal muscle mass index; *BMI*, body mass index; *CRP*, C-reactive protein; *HbA1c*, glycated hemoglobin; *MNA*, mini nutrition assessment; *MMSE*, mini-mental state examination

Variable	Statistic
Age, y	77 (74, 82)
BMI, kg/m^2^	25.0 (22.6, 27.9)
MMSE score, *n* (%)	
Cognitive impairment (< 24 points)	6 (6.3%)
Normal cognition (≥ 24 points)	89 (94%)
MNA score, *n* (%)	
Malnourished (< 17 points)	0 (0%)
At risk of malnutrition (17–23.5 points)	2 (2.1%)
Normal nutritional status (≥ 24 points)	93 (98%)
Comorbidities^‡^	
0	20 (22%)
1	50 (55%)
2	19 (21%)
3	2 (2.2%)
Number of prescription drugs, *n* (%)	
None	22 (23%)
1–2 drugs/day	37 (39%)
3–4 drugs/day	24 (25%)
≥ 5 drugs/day	12 (13%)
Plasma HbA1c, mmol/mol ^‡‡^	40 (38, 43)
Serum CRP, mg/L	1.6 (0.8, 3.0)
Serum total cholesterol (mmol/L)	5.6 (5.0, 6.3)
Leukocytes, × 10^9^/L	6.1 (5.0, 7.1)
Monocytes, × 10^9^/L	0.5 (0.4, 0.5)
Maximum handgrip strength, kg	19.5 (17.7, 22.3)
ASMI, kg/kg/m^2^	0.7 (0.6, 0.7)
Gait speed, m/s	1.2 (1.1, 1.4)

### CIBERSORT and associations with functional muscle health

The selected CIBERSORT-estimated leukocyte proportions associated with differential leukocyte counts, as expected (Online Supplement 7).

Three associations between the muscle variables and the CIBERSORT-estimated leukocyte subset proportions were significant, as displayed in Table 2. These associations were between ASMI and monocytes, gait speed and monocytes, and gait speed and M2 macrophages. A representation of all models is found in Online Supplement 8.

**Table 2:** Models significantly associating the muscle variables (exposures) and CIBERSORT-estimated leukocyte subset proportions (outcomes). The table includes *β* coefficients, *p*-values, confidence intervals, and *R*^2^. Abbreviations: *β*, *β* coefficient; *ASMI*, appendicular skeletal muscle mass index; *BMI*, body mass index; *CI*, confidence interval

Exposure	Outcome	*β*	*p*-value	CI	*R*^2^
ASMI (kg/kg/m^2^)	Monocytes	− 0.206	0.024	− 0.385, − 0.028	0.07
Gait speed (m/s)	Monocytes	− 0.090	0.002	− 0.146, − 0.034	0.11
Gait speed (m/s)	Macrophages M2	− 0.026	0.004	− 0.043, − 0.008	0.09

### WGCNA, associations with functional muscle health, and gene set enrichment analysis

The 35 WGCNA gene clusters contained between 28 and 3101 genes and explained between 30.1 and 48.9% of the variance of the genes they contained (excluding the gray cluster, which expectedly explained very little variance) (Online Supplements 9 and 10).

A total of nine associations between the muscle variables and different WGCNA gene clusters were significant; all of them had maximum handgrip strength as exposure. These significant models are presented in Table 3 and all models can be visualized in Online Supplement 11. The genes driving the associations between maximum handgrip strength and the nine gene clusters are shown in Online Supplement 12.

**Table 3:** Significant models associating maximum handgrip strength (exposure) and the WGCNA gene clusters (outcomes). The table includes *β* coefficients, *p*-values, confidence intervals, and *R*^2^. Abbreviations: *β*, β coefficient; *CI*, confidence interval

Exposure	Outcome	*β*	*p*-value	CI	*R*^2^
Maximum handgrip strength (kg)	Darkmagenta cluster	− 0.006	0.029	− 0.011, − 0.001	0.05
	Darkorange cluster	− 0.006	0.033	− 0.011, 0.000	0.05
	Lightcyan cluster	0.008	0.004	0.003, 0.013	0.09
	Lightgreen cluster	− 0.005	0.047	− 0.011, 0.000	0.04
	Orange cluster	− 0.007	0.014	− 0.012, − 0.001	0.06
	Paleturquoise cluster	0.005	0.047	0.000, 0.011	0.04
	Skyblue cluster	− 0.007	0.013	− 0.012, − 0.001	0.06
	Tan cluster	− 0.007	0.005	− 0.013, − 0.002	0.08
	Yellow cluster	− 0.006	0.019	− 0.011, − 0.001	0.06

**Fig. 2:**
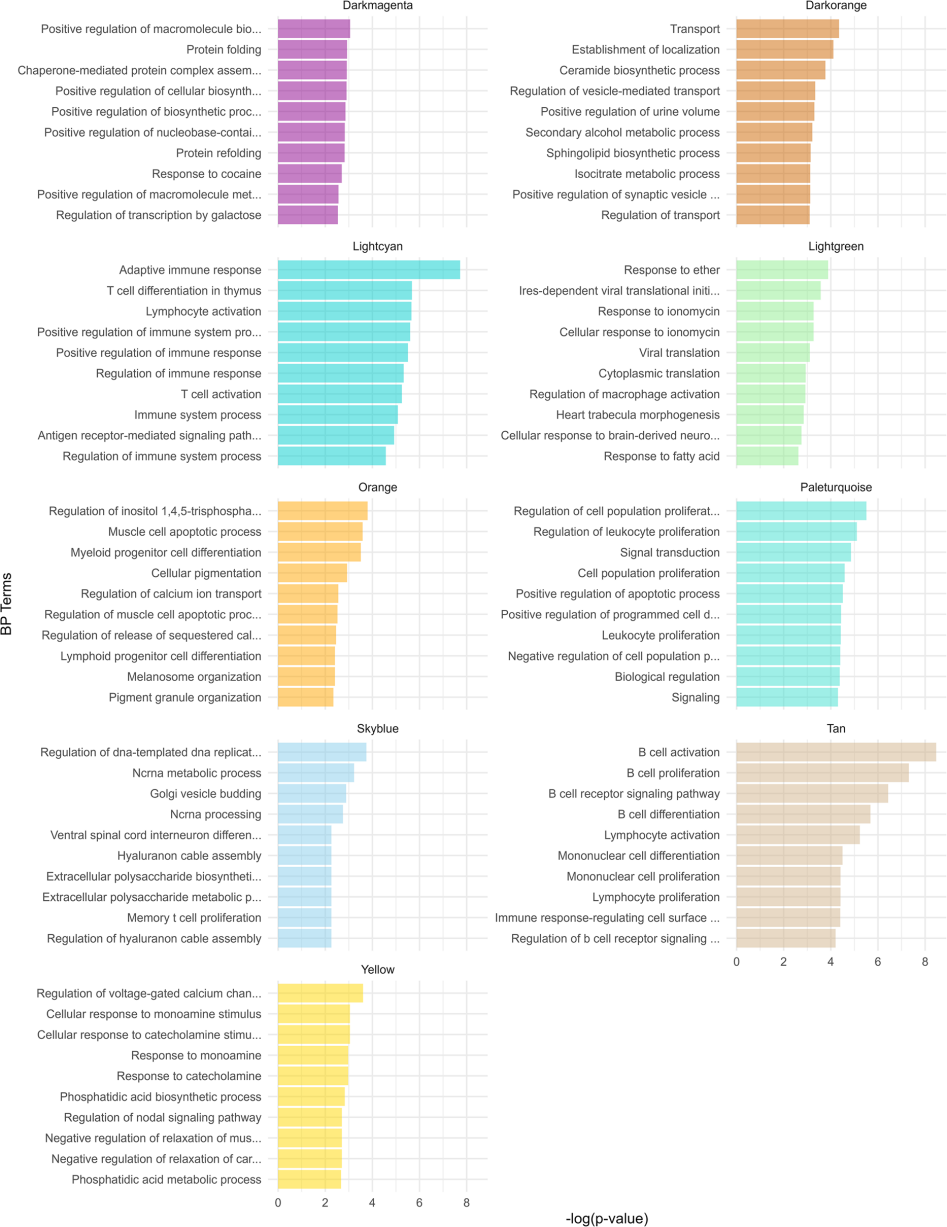
Top 10 enriched BP GO terms for each maximum handgrip strength–associated WGCNA gene cluster. The x-axis indicates − log_10_ of the *p*-value, while the y-axis refers to the BP terms, for each of the gene clusters. Abbreviations: BP, biological process

As seen in Fig. 2 and Online Supplement 13, the gene set enrichment analysis revealed that the nine significant WGCNA gene clusters were enriched in different terms. Of interest, many of the gene clusters were enriched in terms related to many aspects of the immune system (ex: “Leukocyte proliferation” in the Paleturquoise cluster, “B cell activation” in the Tan cluster, “Memory T cell proliferation” in the Skyblue cluster, “Regulation of macrophage activation” in the Lightgreen cluster), intracellular calcium concentration (ex: “Regulation of calcium ion transport” in the Orange cluster, “Regulation of voltage-gated calcium channel activity” in the Yellow cluster), muscle relaxation (ex: “Negative regulation of relaxation of muscle” in the Yellow cluster), and muscle cell apoptosis (ex: “Muscle cell apoptotic process” in the Orange cluster). A sensitivity analysis confirmed the WGCNA gene clusters were enriched in terms related to immune function, while the connection to calcium concentrations and skeletal muscle–related terms were less obvious (Online Supplement 14 and 15).

## Discussion

In this paper, we explored associations between functional muscle health (maximum handgrip strength, ASMI, and gait speed) and PBMC gene expression features (CIBERSORT-estimated leukocyte subset proportions and WGCNA gene clusters), in 95 older women. Our results revealed associations between the individual muscle variables and cells and processes related to the immune system, in addition to suggesting interactions between skeletal muscle cells and circulating PBMCs. Our findings suggest that functional skeletal muscle health may influence circulating PBMCs, raising awareness to a plausible importance of muscle health for the immune system. To the best of our knowledge, this is the first time aging muscle health was explored in relation to proportions of circulating leukocyte subsets and to gene clusters, using whole genome transcriptome data.

### CIBERSORT and associations with functional muscle health

ASMI and gait speed were negatively associated with the estimated circulating monocyte proportions, while gait speed was negatively associated with the estimated circulating M2 macrophage proportions. These results support the involvement of monocytes in functional muscle health, which is plausible, given that increasing age is accompanied by low-grade inflammation [32] and that monocytes play a central role in the inflammatory process [33]. While there are disagreeing reports regarding the numbers of circulating monocytes in older age [34, 35], increased numbers of circulating monocytes have been detected in frail older adults *versus* healthy older participants [17]. In contrast, the association between maximum handgrip strength and the estimated proportion of circulating M2 macrophages is more elusive. Firstly, it is possible that this was a spurious finding, because the detected proportion of M2 macrophages was low, it is unusual for macrophages to be detected in the circulation, and the CIBERSORT algorithm may have identified M2-like monocytes and placed them in the “M2 macrophage” category due to high similarity (the CIBERSORT algorithm does not distinguish between different types of monocytes). Furthermore, it may seem peculiar for physical performance to be negatively associated with circulating amounts of M2 cells, since M2 cells are considered anti-inflammatory [36]. In addition, long-term practice of physical activity has been associated with increased expression of M2-like markers in circulating monocytes, at least in middle-aged women [37]. Therefore, these results must be experimentally confirmed before further hypotheses are formulated.

### WGCNA, associations with functional muscle health, and gene set enrichment analysis

Maximum handgrip strength was associated with nine WGCNA gene clusters. Many of these clusters were enriched in GO biological processes related to immune function and inflammation, calcium signaling, and skeletal muscle cells.

Most processes enriched in the WGCNA gene clusters associated with maximum handgrip strength related to immune function. These findings suggest that functional muscle health may affect immune function, which is plausible. Firstly, in observational studies in older adults, circulating inflammatory markers have been negatively associated with muscle strength, muscle mass [38], and physical performance [39], showing a link between functional skeletal muscle health and immune function. Moreover, mechanistically, skeletal muscle produces cytokines and expresses immune modulatory surface molecules; through these effector molecules, aging skeletal muscle can be hypothesized to aid dysregulating the development and function of different leukocytes and contribute to the establishment of a pro-inflammatory environment (as reviewed in [40]). We also detected a subgroup of processes related to myeloid and lymphoid progenitor cell differentiation. With increasing age, human hematopoietic progenitor cells have their function and quantity altered (reviewed in [41]), possibly assisting the shift towards fewer naïve cells in older adults. Our results suggest that aging skeletal muscle may contribute to this process. These novel findings were supported by a sensitivity analysis and seem to suggest a connection between aging skeletal muscle and immune function.

Furthermore, the WGCNA gene clusters were enriched in a series of other processes that may be indirectly linked to immune function, namely inflammation. For example, we identified terms concerning protein assembly, transport, and localization. These processes may relate to inflammation since accumulated cell “garbage,” such as misfolded proteins and misplaced “self” molecules (such as hyaluronans, another highlighted term) can stimulate an inflammatory response [42]. Another example includes terms related to neurotrophic factors and catecholamines. These terms may be linked to inflammation, as some neurotrophic factors are classified as cytokines (reviewed in [43]) and catecholamines can stimulate monocytes [44]. A final example of processes indirectly related to inflammation is the biosynthesis of ceramides and phosphatidic acid. Circulating ceramide levels have been associated with inflammatory markers in some patient groups [45, 46] and ceramides themselves can stimulate an immune reaction once misplaced (reviewed in [42]). Phosphatidic acid functions as regulator of inflammatory response and it is associated with the secretion of pro-inflammatory cytokines by macrophage-like cells [47].

Finally, we identified processes related to calcium transport and muscle cells enriched in the nine WGCNA gene clusters. Namely, we detected terms linked to the release of calcium to the cytosol, muscle cell relaxation, and muscle cell apoptosis. The detection of skeletal muscle–related terms in PBMCs may be coincidental or it may indicate that PBMCs are exposed to and communicate with skeletal muscle. There is evidence of interactions between skeletal muscle and PBMCs in the literature, with skeletal muscle gene expression profiles being reported to reflect PBMC gene expression [20]. Therefore, the expression of skeletal muscle–related transcripts in PBMCs seems plausible, even though its meaning remains elusive. Cytosolic calcium plays several roles, being a second messenger in many cellular contexts (as reviewed in [48]) and being essential for the apoptotic pathway (reviewed in [49]) and for muscle contraction [48]. Muscle strength is the total force generated by the contraction of muscle fibers [50]; consequently, muscle contraction and relaxation processes understandably associate with muscle strength. Furthermore, age-related skeletal muscle changes have been linked to muscle cell apoptosis (as reviewed in [49]). Specifically, dysfunctional cytosolic calcium regulation in skeletal muscle occurs with aging and it may start an apoptotic cascade [49]. Apoptosis may itself be initiated by inflammatory signals in skeletal muscle [49]. Once more, our findings point towards a link between aging skeletal muscle and immune function. This link was not supported by our sensitivity analysis, highlighting the need for further exploration.

### Strengths and limitations

As far as we know, this is the first time that individual, functional, skeletal muscle variables have been associated with proportions of circulating leukocyte subsets and to clusters of highly interconnected genes, in PBMCs. We studied muscle strength, muscle mass, and physical performance separately, even though they are typically analyzed together as part of age-related conditions, such as sarcopenia or frailty syndrome. This may help explain the different results obtained for each of the muscle variables, possibly suggesting that they entail different physiological processes. Our approach and our findings are relevant, as the use of whole genome gene expression dimensionality reduction algorithms gave us a representation of the biological phenomena behind age-related functional muscle health. In addition, this work led us to new hypothesis-generating results to be tested in future studies.

Nonetheless, our study also has limitations. Our participants were generally healthy for their advanced age; even though we did not assess physical activity levels or types of medication taken, they had median normal weight, median HbA1c below the diabetic threshold, good cognitive and nutritional status, low prevalence of comorbidities, low number of prescription drugs taken, and most of our participants had good functional muscle health. Therefore, these results may not be applicable to the general older adult population with worse health status. Another limitation of our study was the narrow age range of our participants, which did not allow us to conclude on the effects of progressing age on the studied gene expression features. It must also be noted that specific genes and gene variants, some related to immune function, are associated to muscle health and they were not assessed in the present work (reviewed in [51]). In addition, our methodology may have faults: for example, CIBERSORT depends on reference profiles [52] for which there are no gold standards, WGCNA assumes suitable pre-processing and normalization of the gene expression data [31], and GO information is permanently incomplete and possibly skewed (reviewed in [53]). CIBERSORT and WGCNA are powerful tools that reduce highly dimensional gene expression data in a biologically plausible way, severely ameliorating multiple testing burden. However, we acknowledge that several tests were performed and multiple testing may still be an issue.

Lastly, our findings must be not be over-interpreted, since they are solely based on the analysis of cross-sectional data and we cannot conclude on causation. Future studies must be carried out to confirm these associations between skeletal muscle and PBMCs. Moreover, if subtle, real associations exist between functional muscle health and PBMC gene expression features, our study likely did not include enough participants to uncover them (as exemplified by the *ad hoc* calculations of sample size for the general linear model using 80% power, in Online Supplement 14). Therefore, especially given that low statistical power increases the probability of false negative and false positive results (as reviewed in [54]), our findings must be interpreted with caution and explored experimentally. The associations identified in the present study would be better understood through a randomized controlled trial with the aim of improving functional muscle health via exercise training, investigating transcriptome changes in skeletal muscle cells and PBMCs, alongside circulating inflammatory cytokines.

### Concluding remarks

In this study, using data from 95 healthy older home-dwelling women, we explored associations between three individual variables indicative of functional muscle health (maximum handgrip strength, ASMI, and gait speed) and two groups of bioinformatics-generated PBMC gene expression features (CIBERSORT-estimated leukocyte subset proportions and WGCNA gene clusters). We detected associations between gait speed and ASMI and the estimated monocyte proportions, and between gait speed and the estimated M2 monocyte proportions. We also detected associations between maximum handgrip strength and nine WGCNA gene clusters, enriched in processes related to immune function and skeletal muscle cells, suggesting a link between skeletal muscle and immune function.

### Supplementary Information

Below is the link to the electronic supplementary material.

Supplementary file1 (PDF 958 KB)

Supplementary file2 (XLSX 46 KB)

## Data Availability

The data that support the findings of this study are not openly available due to reasons of sensitivity and are available from the corresponding author upon reasonable request. Data are located in controlled access data storage at University of Oslo.
