# Supplementary material for: Associations between PBMC whole genome transcriptome, muscle strength, muscle mass, and physical performance in healthy home-dwelling older women
Source: GeroScience. 2023 May 19;45(6):3175–86. doi: 10.1007/s11357-023-00819-0 (PMC10643614; doi:10.1007/s11357-023-00819-0)
Supplement: Supplementary file 1 — Supplementary file1 (PDF 958 KB) [file 11357_2023_819_MOESM1_ESM.pdf]

Online supplements
GeroScience

Associations between PBMC whole genome transcriptome, muscle strength, muscle mass, and physical performance in healthy home-dwelling older women

Ana R. S. Sousa[1], Inger Ottestad [1,2], Gyrd O. Gjevestad[1,3], Kirsten B. Holven[1,4], Stine M. Ulven[1], Jacob J. Christensen[1, 4]

[1]Department of Nutrition, Institute of Basic Medical Sciences, University of Oslo, Sognsvannsveien 9, 0372 Oslo, Norway

[2]The Clinical Nutrition Outpatient Clinic, Section of Clinical Nutrition, Department of Clinical Service, Division of Cancer Medicine, Oslo University Hospital, Sognsvannsveien 20, 0372 Oslo, Norway

[3]TINE SA, Innovation and marketing, Postboks 113 Kalbakken, 0902 Oslo, Norway

[4]Norwegian National Advisory Unit on Familial Hypercholesterolemia, Department of Endocrinology, Morbid Obesity and Preventive Medicine, Oslo University Hospital, Forskningsveien 2B, 0373 Oslo, Norway

Corresponding author:

Jacob J. Christensen, j.j.christensen@medisin.uio.no, Department of Nutrition, Institute of Basic Medical Sciences, University of Oslo, Sognsvannsveien 9, 0372 Oslo, Norway

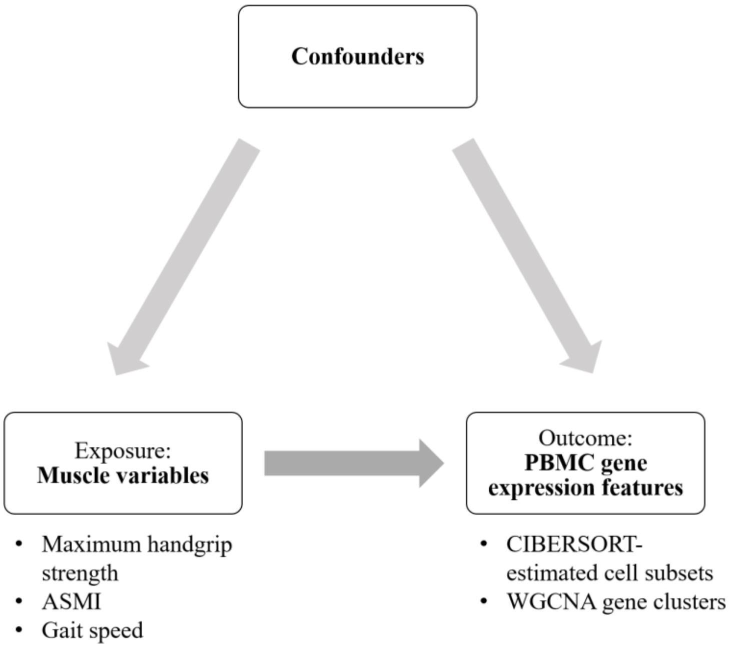

Online Supplement 1 - Directed acyclic graph informative of the linear regression models. The models were made with the muscle variables (maximum handgrip strength, ASMI, and gait speed) as exposures and the PBMC gene expression features (CIBERSORT-estimated leukocyte subset proportions and WGCNA gene clusters) as outcomes. The models involving CIBERSORT-estimated leukocyte subset proportions were adjusted for age and BMI. The models involving WGCNA gene clusters were adjusted for age, BMI, and percentage of monocytes and lymphocytes before running the WGCNA algorithm, using the residual method.

Abbreviations: ASMI: appendicular skeletal muscle mass index, CIBERSORT: cell-type identification by estimating relative subsets of RNA transcripts, WGCNA: weighted correlation network analysis.

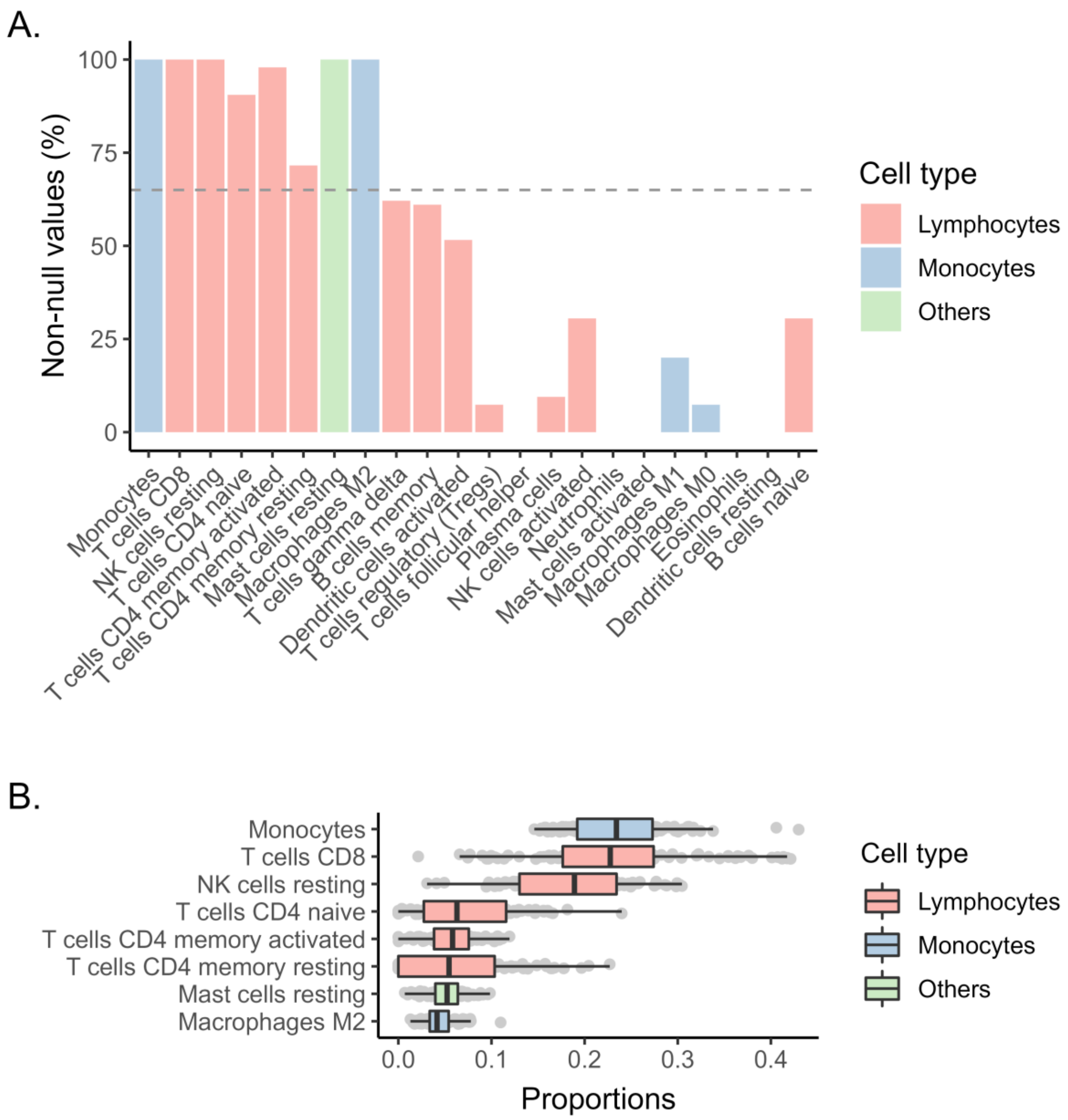

Online Supplement 2 - CIBERSORT-estimated leukocyte subsets. A. Percentage of non-null values for each of the 22 leukocyte subsets identified by CIBERSORT; B. Estimated proportions of the eight leukocyte subsets identified by CIBERSORT with at least 65% non-null values.

Abbreviations: CD: cluster of differentiation, NK: natural killer.

Variable	Statistic
**Clinical variables**	
Age (y)	77 (74, 82)
Weight (kg)	64.4 (57.6, 72.7)
Height (cm)	160 (157, 166)
BMI (kg/m²)	25.0 (22.6, 27.9)
Number of participants > 30 kg/m² (n (%))	12 (12.6 %)
Number of participants > 35 kg/m² (n (%))	5 (5.3 %)
Fat (%)	36.1 (32.3, 40.5)
Fat mass (kg)	23.0 (19.2, 29.2)
FFM (kg)	41.5 (38.8, 44.2)
MMSE score (n (%))	
Cognitive impairment (< 24 points)	6 (6.3 %)
Normal cognition (≥ 24 points)	89 (94 %)
MNA score (n (%))	
Malnourished (< 17 points)	0 (0 %)
At risk of malnutrition (17-23.5 points)	2 (2.1 %)
Normal nutritional status (≥ 24 points)	93 (98 %)
Comorbidities ‡	
0	20 (22 %)
1	50 (55 %)
2	19 (21 %)
3	2 (2.2 %)
Number of prescription drugs (n (%))	
None	22 / 95 (23 %)
1-2 drugs/day	37 / 95 (39 %)
3-4 drugs/day	24 / 95 (25 %)
≥ 5 drugs/day	12 / 95 (13 %)
**Biochemical variables**	
Plasma HbA1c (mmol/mol) ‡‡	40 (38, 43)
Number of participants ≥ 48 mmol/mol (n (%))	4 (4.2 %)
Serum vitamin D (nmol/L)	87 (71, 102)
Number of participants < 50 or > 150 nmol/L (n (%))	4 (4.2 %)
Serum CRP (mg/L)	1.6 (0.8, 3.0)
Number of participants ≥ 5.0 mg/L (n (%))	6 (6.3 %)
Serum Hb (g/100 mL)	13.5 (13.0, 14.1)
Number of participants < 11.7 or > 15.3 g/100 mL (n (%))	4 (4.2 %)
Serum ferritin (µg/L) ‡	112 (68, 169)
Number of participants < 15 or > 200 µg/L (n (%))	17 (17.9 %)

Serum total cholesterol (mmol/L)	5.6 (5.0, 6.3)
Number of participants < 3.9 or > 7.8 mmol/L (n (%))	4 (4.2 %)
Serum LDL cholesterol (mmol/L)	3.2 (2.6, 3.8)
Number of participants < 2.0 or > 5.3 mmol/L (n (%))	7 (7.4 %)
Serum HDL cholesterol (mmol/L)	1.8 (1.5, 2.1)
Number of participants < 1.0 or > 2.7 mmol/L (n (%))	6 (6.3 %)
Serum TG (mmol/L)	1.40 (1.02, 1.79)
Number of participants > 4.00 mmol/L (n (%))	2 (2.1 %)
Leukocytes (x 10^9/L)	6.1 (5.0, 7.1)
Number of participants < 3.5 or > 10.0 x 10^9/L (n (%))	3 (3.2 %)
Neutrophils (x 10^9/L)	3.5 (2.7, 4.2)
Number of participants <1.5 or > 7.3 x 10^9/L (n (%))	1 (1.1 %)
Neutrophils (%)	57.7 (50.9, 64.2)
Number of participants < 38.0 or > 71.0 % (n (%))	9 (9.5 %)
Lymphocytes (x 10^9/L)	1.8 (1.5, 2.2)
Number of participants < 1.1 or > 3.3 x 10^9/L (n (%))	5 (5.3 %)
Lymphocytes (%)	31.1 (24.5, 36.4)
Number of participants < 20.0 or > 50.0 (n (%))	10 (10.5 %)
Monocytes (x 10^9/L)	0.5 (0.4, 0.5)
Number of participants < 0.2 or > 0.8 x 10^9/L (n (%))	5 (5.3 %)
Monocytes (%)	7.8 (6.9, 9.4)
Number of participants < 5.0 or 14.0 % (n (%))	4 (4.2 %)
Eosinophils (x 10^9/L)	0.1 (0.1, 0.2)
Number of participants ≥ 0.4 x 10^9/L (n (%))	2 (2.1 %)
Eosinophils (%)	2.1 (1.6, 3.6)
Number of participants ≥ 7.0 % (n (%))	2 (2.1 %)
**Muscle variables**	
Maximum handgrip strength (kg)	19.5 (17.7, 22.3)
Number of participants < 16.0 kg (n (%))	8 (8.4 %)
ASMI (kg/kg/m²)	0.7 (0.6, 0.7)
Number of participants < 0.512 kg/kg/m² (n (%))	2 (2.1 %)
Gait speed (m/s)	1.2 (1.1, 1.4)
Number of participants ≤ 0.8 m/s (n (%))	6 (6.3 %)

Online Supplement 3 – Extended characteristics of the study population. For continuous variables, the values are medians (p25, p75); for categorical variables, the values are n/N. Comorbidities include history of cancer, cardiovascular disease, hypertension, respiratory disease, severe inflammatory disease and/or type 2 diabetes. Basophile count not shown; 62 participants (65 %) had a basophil count of zero, 17 participants (18 %) had a count of 0.1 x 10^9/L, and 62 (65 %) had < 0.1 x 10^9/L.

‡ four missing values; ‡‡ five missing values.

Abbreviations: ASMI: appendicular skeletal muscle mass index; BMI: body mass index; CRP: C-reactive protein; FFM: fat-free mass; Hb: hemoglobin; HbA1c: glycated hemoglobin; HDL: high density lipoprotein; LDL: low density lipoprotein; MNA: mini nutrition assessment; MMSE: mini-mental state examination; TG: triglycerides.

Variable	First quartile	Second quartile	Third quartile	Fourth quartile	p-value
**Clinical variables**					
Age (y)	82 (77, 84)	76 (73, 80)	76 (74, 80)	74 (74, 81)	0.056
Weight (kg)	59.2 (56.8, 67.1)	61.5 (57.2, 71.3)	66.8 (62.6, 74.4)	69.0 (59.8, 74.6)	0.058
Height (cm)	159 (156, 163)	159 (156, 164)	160 (158, 166)	163 (160, 168)	0.059
BMI (kg/m^2)	23.2 (22.7, 25.8)	25.3 (22.1, 27.3)	26.2 (23.8, 28.2)	25.8 (21.6, 29.4)	0.2
Fat (%)	35.5 (32.0, 40.0)	36.5 (32.3, 39.0)	37.7 (33.6, 40.7)	34.9 (30.0, 41.8)	0.8
Fat mass (kg)	21.6 (17.4, 25.4)	23.5 (18.9, 27.5)	25.0 (21.1, 30.7)	26.4 (18.9, 31.8)	0.4
FFM (kg)	40.1 (37.3, 40.9)	40.4 (38.3, 43.7)	42.9 (40.8, 44.9)	42.7 (41.6, 46.1)	0.001
MMSE score					0.078
<24 points	3 / 24 (12%)	0 / 24 (0%)	3 / 24 (12%)	0 / 23 (0%)	
≥24 points	21 / 24 (88%)	24 / 24 (100%)	21 / 24 (88%)	23 / 23 (100%)	
MNA score					0.6
Malnourished	0 / 24 (0%)	0 / 24 (0%)	0 / 24 (0%)	0 / 23 (0%)	
At risk of malnutrition	0 / 24 (0%)	1 / 24 (4.2%)	0 / 24 (0%)	1 / 23 (4.3%)	
Not malnourished	24 / 24 (100%)	23 / 24 (96%)	24 / 24 (100%)	22 / 23 (96%)	
Comorbidities [‡]					0.078
No comorbidities	2 / 23 (8.7%)	8 / 24 (33%)	4 / 23 (17%)	6 / 21 (29%)	
One comorbidity	18 / 23 (78%)	11 / 24 (46%)	10 / 23 (43%)	11 / 21 (52%)	
Two comorbidities	2 / 23 (8.7%)	4 / 24 (17%)	9 / 23 (39%)	4 / 21 (19%)	
Three comorbidities	1 / 23 (4.3%)	1 / 24 (4.2%)	0 / 23 (0%)	0 / 21 (0%)	
Number of prescription drugs					0.7
None	4 / 24 (17%)	8 / 24 (33%)	3 / 24 (12%)	7 / 23 (30%)	
1-2 drugs/day	10 / 24 (42%)	8 / 24 (33%)	11 / 24 (46%)	8 / 23 (35%)	
3-4 drugs/day	8 / 24 (33%)	6 / 24 (25%)	6 / 24 (25%)	4 / 23 (17%)	
≥5 drugs/day	2 / 24 (8.3%)	2 / 24 (8.3%)	4 / 24 (17%)	4 / 23 (17%)	
**Biochemical variables**					
Plasma HbA1c (mmol/mol) [‡‡]	40 (37, 43)	40 (38, 42)	40 (39, 45)	41 (39, 43)	0.5
Serum vitamin D (nmol/L)	94 (74, 112)	86 (72, 100)	74 (68, 92)	92 (74, 100)	0.092
Serum CRP (mg/L)	2.2 (0.9, 3.8)	1.6 (0.7, 2.5)	1.8 (0.8, 3.0)	1.3 (0.7, 2.2)	0.5
Serum Hb (g/100 mL)	13.6 (12.9, 14.1)	13.4 (13.0, 13.8)	13.6 (13.1, 14.2)	13.5 (13.0, 14.1)	0.8
Serum ferritin (μg/L) [‡]	121 (62, 146)	112 (77, 158)	94 (66, 187)	113 (72, 165)	>0.9
Serum total cholesterol (mmol/L)	5.8 (5.2, 6.5)	5.3 (5.0, 6.2)	5.8 (5.3, 6.1)	5.4 (4.8, 6.6)	0.6
Serum LDL cholesterol (mmol/L)	3.2 (2.6, 4.2)	3.2 (2.7, 3.7)	3.5 (2.5, 3.8)	3.1 (2.6, 3.4)	0.9
Serum HDL cholesterol (mmol/L)	1.9 (1.6, 2.3)	1.8 (1.5, 1.9)	1.8 (1.5, 2.1)	1.6 (1.5, 2.2)	0.9
Serum TG (mmol/L)	1.24 (0.98, 1.76)	1.38 (1.13, 1.78)	1.55 (1.13, 1.85)	1.33 (1.02, 1.80)	0.8
Leukocytes (x 10^9/L)	6.7 (5.3, 7.6)	6.2 (5.3, 7.0)	5.9 (5.0, 7.0)	5.8 (4.6, 6.8)	0.3
Neutrophils (x 10^9/L)	3.5 (3.0, 5.0)	3.6 (3.0, 4.2)	3.5 (2.6, 4.0)	3.1 (2.7, 3.8)	0.2

Neutrophils (%)	59.2 (51.6, 68.1)	60.2 (52.9, 64.3)	54.1 (49.8, 61.2)	55.7 (50.4, 63.9)	0.3
Lymphocytes (x 10^9/L)	1.8 (1.6, 2.1)	1.9 (1.5, 2.1)	1.8 (1.7, 2.0)	1.7 (1.3, 2.3)	0.8
Lymphocytes (%)	29.4 (20.6, 34.2)	30.4 (25.5, 35.5)	32.9 (28.8, 37.3)	34.0 (25.3, 37.0)	0.2
Monocytes (x 10^9/L)	0.5 (0.5, 0.7)	0.5 (0.4, 0.5)	0.5 (0.4, 0.5)	0.5 (0.5, 0.6)	0.10
Monocytes (%)	8.3 (6.7, 9.7)	7.5 (6.2, 8.8)	7.7 (6.9, 8.2)	8.6 (7.4, 9.9)	0.091
Eosinophils (x 10^9/L)	0.2 (0.1, 0.2)	0.1 (0.1, 0.2)	0.1 (0.1, 0.2)	0.1 (0.1, 0.2)	0.6
Eosinophils (%)	2.4 (1.5, 3.6)	1.9 (1.5, 3.0)	2.6 (1.7, 3.7)	2.2 (1.6, 2.9)	0.8
**Muscle variables**					
Maximum handgrip strength (kg)	16.2 (14.9, 17.0)	19.0 (18.3, 19.3)	21.2 (20.5, 21.7)	24.9 (23.7, 26.4)	<0.001
ASMI (kg/kg/m²)	0.7 (0.6, 0.7)	0.7 (0.6, 0.7)	0.7 (0.6, 0.7)	0.7 (0.7, 0.8)	0.072
Gait speed (m/s)	1.11 (0.92, 1.19)	1.28 (1.09, 1.40)	1.23 (1.12, 1.42)	1.22 (1.15, 1.35)	0.029

Online Supplement 4 – Characteristics of the study population by quartiles of maximum handgrip strength. For continuous variables, the values are medians (p25, p75); for categorical variables, the values are n/N. P-values acquired using the Kruskal-Wallis test for continuous variables and the Chi-Squared test when all expected cell counts ≥5 or the Fisher test when any expected cell count <5, for categorical variables. Comorbidities include history of cancer, cardiovascular disease, hypertension, respiratory disease, severe inflammatory disease and/or type 2 diabetes.

‡ four missing values; ‡‡ five missing values.

Variable	First quartile	Second quartile	Third quartile	Fourth quartile	p-value
**Clinical variables**					
Age (y)	77 (74, 82)	79 (74, 81)	77 (74, 84)	79 (74, 82)	>0.9
Weight (kg)	69.2 (64.7, 77.0)	67.5 (57.6, 75.2)	62.6 (58.6, 72.5)	58.2 (54.1, 63.8)	0.004
Height (cm)	156 (154, 159)	160 (156, 164)	164 (160, 166)	166 (160, 168)	<0.001
BMI (kg/m²)	27.4 (26.4, 31.0)	25.9 (23.8, 28.8)	23.6 (22.9, 25.7)	21.7 (20.6, 23.0)	<0.001
Fat (%)	41.4 (37.8, 44.0)	36.8 (34.2, 41.0)	35.4 (32.6, 38.2)	29.6 (24.8, 32.9)	<0.001
Fat mass (kg)	28.4 (24.3, 33.5)	24.8 (20.5, 29.9)	22.0 (19.3, 28.0)	18.1 (13.7, 21.2)	<0.001
FFM (kg)	40.9 (38.5, 43.5)	42.0 (37.9, 45.1)	41.4 (39.4, 44.7)	41.9 (39.7, 43.2)	0.9
MMSE score					0.4
<24 points	3 / 24 (12%)	0 / 24 (0%)	2 / 24 (8.3%)	1 / 23 (4.3%)	
≥24 points	21 / 24 (88%)	24 / 24 (100%)	22 / 24 (92%)	22 / 23 (96%)	
MNA score					0.6
Malnourished	0 / 24 (0%)	0 / 24 (0%)	0 / 24 (0%)	0 / 23 (0%)	
At risk of malnutrition	1 / 24 (4.2%)	0 / 24 (0%)	0 / 24 (0%)	1 / 23 (4.3%)	
Not malnourished	23 / 24 (96%)	24 / 24 (100%)	24 / 24 (100%)	22 / 23 (96%)	
Comorbidities[‡]					0.4
No comorbidities	7 / 23 (30%)	3 / 23 (13%)	5 / 23 (22%)	5 / 22 (23%)	
One comorbidity	13 / 23 (57%)	12 / 23 (52%)	15 / 23 (65%)	10 / 22 (45%)	
Two comorbidities	3 / 23 (13%)	6 / 23 (26%)	3 / 23 (13%)	7 / 22 (32%)	
Three comorbidities	0 / 23 (0%)	2 / 23 (8.7%)	0 / 23 (0%)	0 / 22 (0%)	
Number of prescription drugs					0.9
None	5 / 24 (21%)	5 / 24 (21%)	5 / 24 (21%)	7 / 23 (30%)	
1-2 drugs/day	10 / 24 (42%)	10 / 24 (42%)	9 / 24 (38%)	8 / 23 (35%)	
3-4 drugs/day	5 / 24 (21%)	5 / 24 (21%)	9 / 24 (38%)	5 / 23 (22%)	
≥5 drugs/day	4 / 24 (17%)	4 / 24 (17%)	1 / 24 (4.2%)	3 / 23 (13%)	
**Biochemical variables**					
Plasma HbA1c (mmol/mol) [‡‡]	42 (40, 44)	39 (38, 42)	39 (37, 41)	40 (40, 42)	0.026
Serum vitamin D (nmol/L)	80 (65, 96)	96 (87, 105)	83 (71, 104)	87 (74, 98)	0.12
Serum CRP (mg/L)	3.2 (1.8, 4.2)	1.9 (1.1, 2.7)	1.5 (0.9, 2.0)	0.8 (0.7, 1.6)	<0.001
Serum Hb (g/100 mL)	13.8 (13.4, 14.2)	13.6 (13.0, 14.1)	13.4 (12.8, 14.0)	13.3 (12.9, 13.8)	0.13
Serum ferritin (µg/L) [‡]	155 (92, 210)	106 (73, 127)	107 (61, 161)	93 (62, 143)	0.13
Serum total cholesterol (mmol/L)	5.5 (5.0, 5.9)	5.4 (5.0, 6.2)	5.4 (5.0, 6.2)	5.9 (5.3, 6.7)	0.5
Serum LDL cholesterol (mmol/L)	3.1 (2.5, 3.7)	3.2 (2.6, 4.1)	3.2 (2.5, 3.7)	3.1 (2.7, 3.7)	>0.9
Serum HDL cholesterol (mmol/L)	1.7 (1.5, 1.8)	1.7 (1.4, 2.2)	1.8 (1.5, 2.2)	2.0 (1.7, 2.2)	0.13
Serum TG (mmol/L)	1.67 (1.28, 1.96)	1.50 (1.14, 1.87)	1.25 (0.87, 1.47)	1.24 (1.04, 1.74)	0.027
Leukocytes (x 10^9/L)	6.6 (4.9, 7.2)	5.8 (5.2, 7.1)	6.2 (5.2, 7.2)	5.5 (4.8, 6.8)	0.8
Neutrophils (x 10^9/L)	3.5 (2.8, 4.1)	3.7 (2.8, 4.7)	3.4 (2.8, 4.0)	3.0 (2.7, 4.2)	0.8
Neutrophils (%)	56.7 (50.8, 62.2)	60.8 (54.0, 70.7)	54.3 (49.8, 62.2)	58.3 (51.3, 65.1)	0.2

Lymphocytes (x 10^9/L)	2.0 (1.7, 2.2)	1.7 (1.4, 1.9)	1.8 (1.7, 2.1)	1.7 (1.3, 2.2)	0.2
Lymphocytes (%)	31.2 (27.8, 35.8)	29.4 (20.9, 32.5)	34.0 (24.4, 38.8)	32.1 (26.3, 36.5)	0.2
Monocytes (x 10^9/L)	0.5 (0.4, 0.5)	0.5 (0.4, 0.6)	0.5 (0.4, 0.6)	0.5 (0.4, 0.5)	0.6
Monocytes (%)	7.6 (6.4, 8.4)	8.5 (7.1, 9.6)	7.9 (7.1, 10.0)	7.6 (6.9, 8.3)	0.4
Eosinophils (x 10^9/L)	0.1 (0.1, 0.3)	0.1 (0.1, 0.2)	0.1 (0.1, 0.2)	0.1 (0.1, 0.2)	0.3
Eosinophils (%)	2.5 (1.6, 4.4)	1.9 (1.5, 2.9)	2.2 (1.7, 3.7)	2.2 (1.5, 3.0)	0.3
**Muscle variables**					
Maximum handgrip strength (kg)	19.7 (18.8, 22.1)	18.2 (16.5, 20.6)	19.8 (18.2, 21.9)	21.8 (18.9, 24.5)	0.032
ASMI (kg/kg/m²)	0.6 (0.6, 0.6)	0.7 (0.6, 0.7)	0.7 (0.7, 0.7)	0.8 (0.7, 0.8)	<0.001
Gait speed (m/s)	1.23 (1.13, 1.39)	1.12 (0.92, 1.32)	1.17 (1.09, 1.33)	1.24 (1.16, 1.40)	0.069

Online Supplement 5 – Characteristics of the study population by quartiles of ASMI. For continuous variables, the values are medians (p25, p75); for categorical variables, the values are n/N. P-values acquired using the Kruskal-Wallis test for continuous variables and the Chi-Squared test when all expected cell counts ≥5 or the Fisher test when any expected cell count <5, for categorical variables. Comorbidities include history of cancer, cardiovascular disease, hypertension, respiratory disease, severe inflammatory disease and/or type 2 diabetes.

‡ four missing values; ‡‡ five missing values.

Variable	First quartile	Second quartile	Third quartile	Fourth quartile	p-value
**Clinical variables**					
Age (y)	81 (79, 87)	80 (74, 83)	79 (73, 81)	74 (73, 75)	<0.001
Weight (kg)	62.0 (57.5, 72.5)	66.7 (58.7, 73.6)	65.0 (57.6, 71.4)	63.1 (55.3, 71.5)	0.8
Height (cm)	160 (158, 162)	160 (157, 166)	160 (156, 167)	161 (158, 166)	0.9
BMI (kg/m^2)	25.0 (22.8, 28.3)	25.2 (23.4, 29.2)	25.8 (22.4, 27.4)	23.8 (21.1, 27.2)	0.8
Fat (%)	36.4 (34.0, 40.0)	38.2 (33.7, 41.9)	35.0 (31.8, 39.0)	32.8 (30.0, 38.7)	0.4
Fat mass (kg)	23.5 (20.5, 28.6)	24.9 (20.0, 31.4)	22.9 (18.8, 28.9)	21.5 (17.8, 27.6)	0.6
FFM (kg)	40.7 (38.2, 44.1)	41.4 (40.0, 44.4)	41.6 (38.8, 43.9)	42.0 (40.1, 44.1)	>0.9
MMSE score					0.9
<24 points	2 / 24 (8.3%)	1 / 24 (4.2%)	1 / 24 (4.2%)	2 / 23 (8.7%)	
≥24 points	22 / 24 (92%)	23 / 24 (96%)	23 / 24 (96%)	21 / 23 (91%)	
MNA score					0.2
Malnourished	0 / 24 (0%)	0 / 24 (0%)	0 / 24 (0%)	0 / 23 (0%)	
At risk of malnutrition	2 / 24 (8.3%)	0 / 24 (0%)	0 / 24 (0%)	0 / 23 (0%)	
Not malnourished	22 / 24 (92%)	24 / 24 (100%)	24 / 24 (100%)	23 / 23 (100%)	
Comorbidities[‡]					0.5
No comorbidities	4 / 23 (17%)	3 / 23 (13%)	6 / 23 (26%)	7 / 22 (32%)	
One comorbidity	11 / 23 (48%)	16 / 23 (70%)	11 / 23 (48%)	12 / 22 (55%)	
Two comorbidities	6 / 23 (26%)	4 / 23 (17%)	6 / 23 (26%)	3 / 22 (14%)	
Three comorbidities	2 / 23 (8.7%)	0 / 23 (0%)	0 / 23 (0%)	0 / 22 (0%)	
Number of prescription drugs					0.10
None	3 / 24 (12%)	5 / 24 (21%)	8 / 24 (33%)	6 / 23 (26%)	
1-2 drugs/day	6 / 24 (25%)	9 / 24 (38%)	11 / 24 (46%)	11 / 23 (48%)	
3-4 drugs/day	11 / 24 (46%)	4 / 24 (17%)	4 / 24 (17%)	5 / 23 (22%)	
≥5 drugs/day	4 / 24 (17%)	6 / 24 (25%)	1 / 24 (4.2%)	1 / 23 (4.3%)	
**Biochemical variables**					
Plasma HbA1c (mmol/mol)[‡‡]	40 (38, 42)	41 (38, 44)	41 (38, 45)	41 (39, 42)	0.7
Serum vitamin D (nmol/L)	95 (83, 109)	86 (72, 96)	84 (70, 98)	84 (70, 102)	0.4
Serum CRP (mg/L)	2.1 (0.8, 3.6)	1.3 (0.9, 2.7)	1.5 (0.9, 2.5)	1.7 (0.8, 2.4)	0.9
Serum Hb (g/100 mL)	13.6 (12.5, 13.9)	13.6 (12.9, 14.2)	13.4 (13.1, 14.0)	13.8 (13.2, 14.1)	0.4
Serum ferritin (μg/L)[‡]	121 (70, 144)	162 (83, 186)	110 (66, 174)	84 (51, 124)	0.2
Serum total cholesterol (mmol/L)	5.8 (5.2, 6.2)	5.3 (4.6, 6.2)	5.3 (5.0, 6.0)	5.8 (5.5, 6.7)	0.11
Serum LDL cholesterol (mmol/L)	3.3 (2.5, 4.0)	3.1 (2.2, 3.7)	2.8 (2.6, 3.3)	3.5 (3.2, 4.2)	0.069
Serum HDL cholesterol (mmol/L)	1.7 (1.5, 2.1)	1.6 (1.4, 2.1)	2.0 (1.6, 2.3)	1.8 (1.6, 2.2)	0.5
Serum TG (mmol/L)	1.28 (1.00, 1.81)	1.44 (1.06, 1.93)	1.29 (0.89, 1.78)	1.44 (1.35, 1.63)	0.6
Leukocytes (x 10^9/L)	6.0 (5.4, 7.6)	6.3 (5.3, 7.0)	5.8 (4.7, 6.7)	6.5 (5.0, 7.0)	0.5
Neutrophils (x 10^9/L)	3.6 (3.0, 4.8)	3.5 (2.7, 4.0)	3.1 (2.6, 3.9)	3.2 (2.7, 4.3)	0.6

Neutrophils (%)	58.8 (50.4, 66.8)	58.4 (53.0, 62.7)	56.7 (50.8, 62.7)	56.6 (50.0, 62.3)	0.8
Lymphocytes (x 10^9/L)	1.8 (1.5, 2.1)	1.8 (1.3, 2.0)	1.7 (1.5, 2.2)	1.9 (1.7, 2.2)	0.7
Lymphocytes (%)	28.3 (21.3, 35.5)	29.7 (25.7, 34.7)	31.3 (28.9, 37.9)	33.3 (27.1, 38.0)	0.4
Monocytes (x 10^9/L)	0.5 (0.5, 0.7)	0.5 (0.5, 0.5)	0.4 (0.4, 0.5)	0.5 (0.3, 0.5)	0.004
Monocytes (%)	9.0 (8.5, 10.2)	7.8 (7.1, 8.4)	7.6 (6.5, 9.6)	7.4 (6.3, 7.8)	0.003
Eosinophils (x 10^9/L)	0.1 (0.1, 0.2)	0.1 (0.1, 0.2)	0.1 (0.1, 0.2)	0.1 (0.1, 0.2)	0.9
Eosinophils (%)	2.0 (1.6, 3.5)	2.4 (1.7, 3.6)	2.2 (1.6, 4.0)	2.0 (1.5, 2.8)	0.8
**Muscle variables**					
Maximum handgrip strength (kg)	18.0 (15.9, 19.5)	20.9 (18.0, 24.8)	21.0 (18.6, 23.5)	19.9 (18.8, 21.9)	0.010
ASMI (kg/kg/m²)	0.7 (0.6, 0.7)	0.7 (0.6, 0.7)	0.7 (0.6, 0.7)	0.7 (0.6, 0.8)	0.9
Gait speed (m/s)	0.94 (0.83, 1.05)	1.15 (1.12, 1.17)	1.29 (1.23, 1.32)	1.45 (1.42, 1.50)	<0.001

Online Supplement 6 – Characteristics of the study population by quartiles of gait speed. For continuous variables, the values are medians (p25, p75); for categorical variables, the values are n/N. P-values acquired using the Kruskal-Wallis test for continuous variables and the Chi-Squared test when all expected cell counts ≥5 or the Fisher test when any expected cell count <5, for categorical variables. Comorbidities include history of cancer, cardiovascular disease, hypertension, respiratory disease, severe inflammatory disease and/or type 2 diabetes.

‡ four missing values; ‡‡ five missing values.

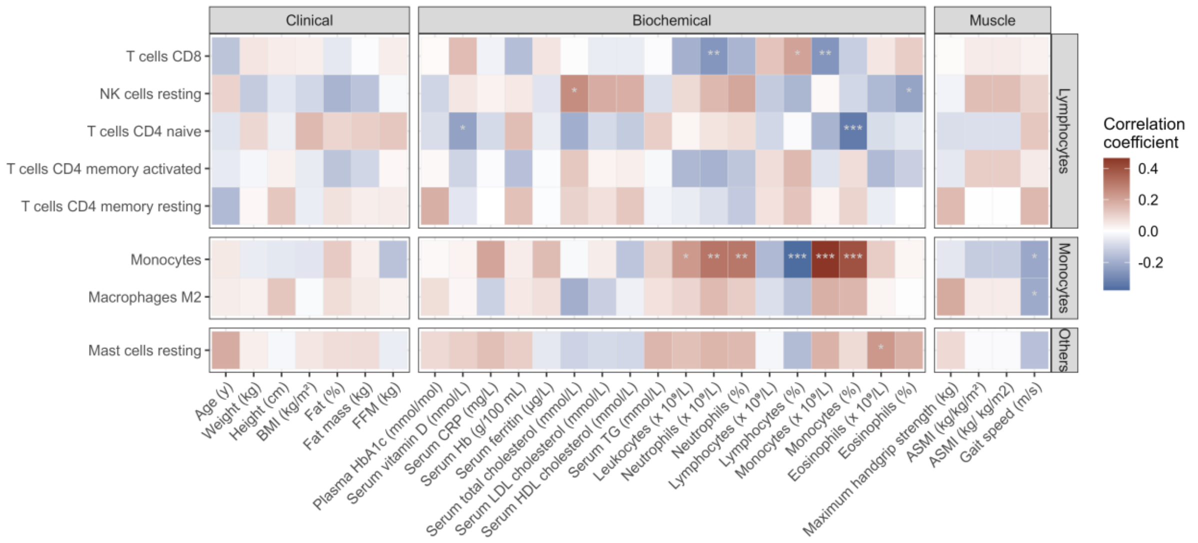

Online Supplement 7 – Correlations between the selected CIBERSORT-estimated leukocyte subset proportions and the clinical, biochemical, and muscle variables.

The figure displays Spearman’s correlation coefficient between the study population variables and the CIBERSORT-estimated leukocyte subsets in the x and y axes, respectively.

* p-value < 0.05, ** p-value < 0.01, *** p-value < 0.001.

Abbreviations: ASMI: appendicular skeletal muscle mass index; BMI: body mass index; CD: cluster of differentiation; CRP: C-reactive protein; FFM: fat-free mass; Hb: hemoglobin; HbA1c: glycated hemoglobin; HDL: high density lipoprotein; LDL: low density lipoprotein; MNA: mini nutrition assessment; MMSE: mini-mental state examination; NK: natural killer; TG: triglycerides.

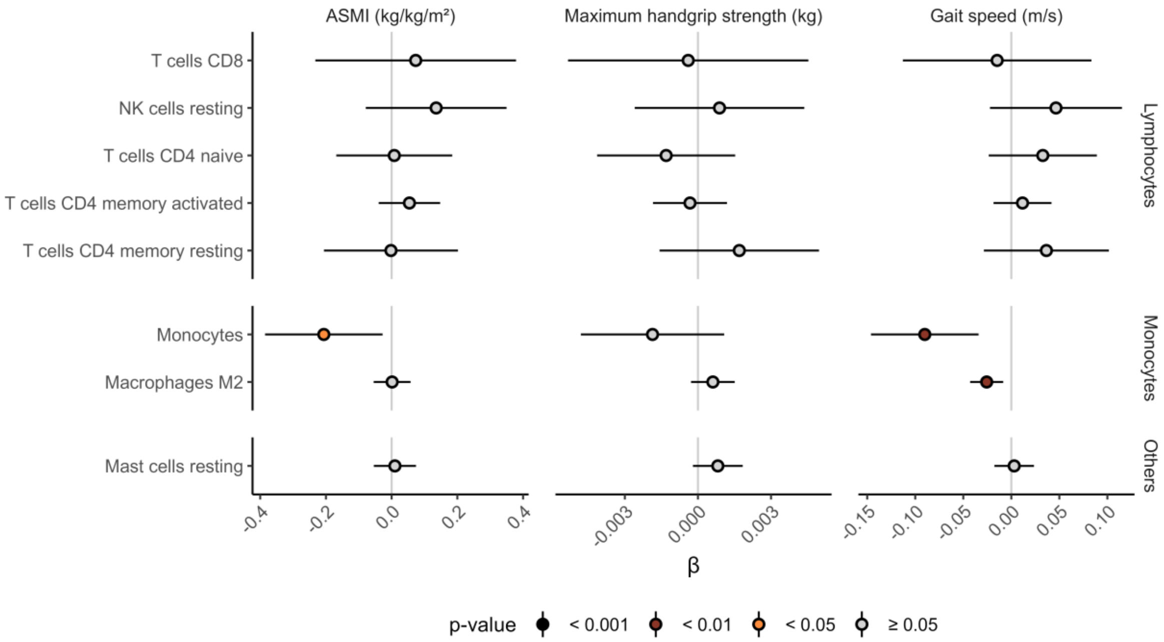

Online Supplement 8 - Forest plot of the β coefficients of the linear regression models associating the muscle variables and the CIBERSORT-estimated leukocyte subset proportions.

The x-axis indicates the β coefficients (95 % CI) for each of the exposures, while the y-axis indicates the leukocyte subsets. The colored dots represent the β coefficients and their p-values; the lateral lines indicate the associated confidence intervals.

Abbreviations: ASMI: appendicular skeletal muscle mass index; CD: cluster of differentiation, NK: natural killer, β: β coefficient.

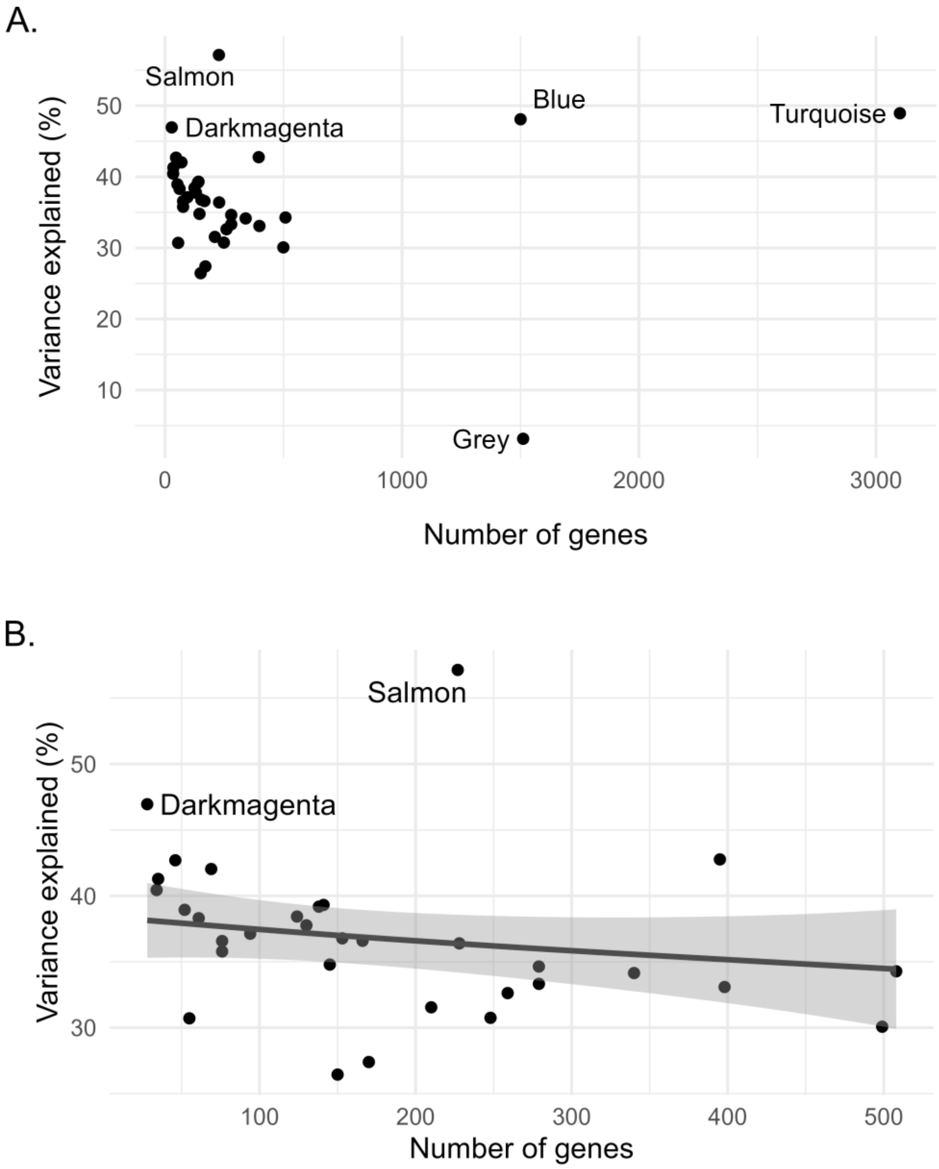

Online Supplement 9 – Gene expression variance explained by the eigengenes (equivalent to principal component 1) versus cluster size. A. All clusters generated by WGCNA; B. Only clusters with fewer than 1000 genes, zoomed in for visibility. The solid line is a generalized additive mode smoothing line, and the grey bands represent standard error.

**Cluster**	**Number of genes**	**Variance explained (%)**
Turquoise	3101	48.9
Grey	1511	3.2
Blue	1500	48.1
Brown	508	34.3
Yellow	499	30.1
Green	398	33.1
Red	395	42.8
Black	340	34.1
Magenta	279	34.6
Pink	279	33.3
Purple	259	32.6
Greenyellow	248	30.7
Tan	228	36.4
Salmon	227	57.1
Cyan	210	31.5
Midnightblue	170	27.4
Lightcyan	166	36.6
Grey60	153	36.8
Lightgreen	150	26.4
Lightyellow	145	34.8
Royalblue	141	39.3
Darkred	138	39.2
Darkgreen	130	37.8
Darkturquoise	124	38.4
Darkgrey	94	37.1
Orange	76	36.6
Darkorange	76	35.8
White	69	42.0
Skyblue	61	38.3
Saddlebrown	55	30.7
Steelblue	52	38.9
Paleturquoise	46	42.7
Violet	35	41.3
Darkolivegreen	34	40.4
Darkmagenta	28	46.9

Online Supplement 10 – Number of genes and percentage variance explained per WGCNA gene cluster.

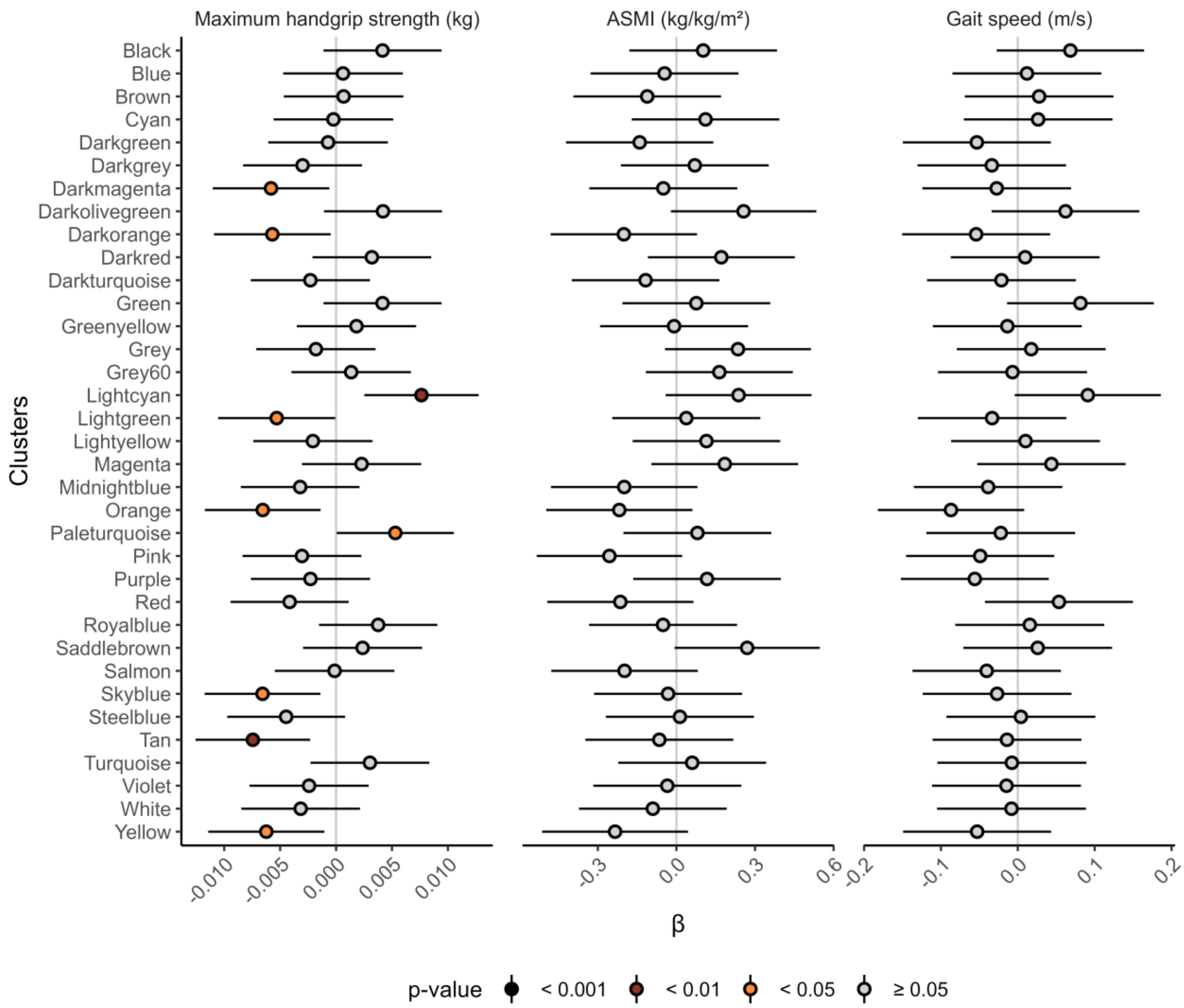

Online Supplement 11 – Forest plot of the β coefficients of the linear regression models associating the muscle variables and the WGCNA gene clusters.

The x-axis indicates the β coefficients (95 % CI) for each of the exposures, while the y-axis indicates the gene clusters. The colored dots represent the β coefficients and their p-values; the lateral lines indicate the associated confidence intervals.

Abbreviations: ASMI: appendicular skeletal muscle mass index; β: β coefficient.

	CIBERSORT 3 numerator degrees of freedom			WGCNA 5 numerator degrees of freedom				
**R^2 / p-value**	**0.07**	**0.09**	**0.11**	**0.04**	**0.05**	**0.06**	**0.08**	**0.09**
**0.05**	149	114	92	313	249	207	153	135
**0.01**	211	162	131	436	347	288	213	188
**0.001**	294	226	182	597	475	393	292	258

Online Supplement 14 – *Ad-hoc* power calculations for the significant models associating the muscle variables and the CIBERSORT-estimated leukocyte subset proportions and the WGCNA gene clusters.

Computed using power calculations for the general linear model, pwr::pwr.f2.test, assuming 80% power, for significance levels of 0.05, 0.01, and 0.001.
